# The Technique of Gaining Entrance to Pulp Chambers and Root Canals

**Published:** 1900-07-15

**Authors:** L. P. Hall


### THE TECHNIQUE OF GAINING ENTRANCE TO PULP CHAMBERS AND ROOT CANALS.

BY L. P. HALL, D.D.S.

In presenting this paper it is not with the hope of bringing out anything new or unexpected. It will discuss the methods of treating teeth only so far as they may affect the opening of cavities or canals. We will suppose that the teeth are already devitalized, and that a rubber dam has been placed on the teeth where possible. If the tooth is very sore we may not need a dam the first time, as all we can probably do will be to give relief, and, perhaps, syringe it out. But when the treatment is begun the rubber dam is certainly needed. The dam saves time, prevents the entrance of things you do not put in yourself, and gives a clean, dry view of the work. No wonder we hear patients say a tooth has been treated for so many months or a year when the operator has poked his treatment through saliva and debris all the time, very likely never having seen the canals or knowing surely how many there are.

There are places where the rubber can not be used. There are cases presented, for instance, in which it is almost impossible to keep the saliva out, because the decay reaches so far beyond the cervical margin. In these cases it is possible to fill that portion at once with a temporary stopping of cement, amalgam, or gutta-percha, and make a new opening through the crown for treatments. In that way only one tooth need be covered, and that with ease, and no time lost.

Frequently there are patients who can be relied on to keep a cavity dry for change of treatment, but in most cases it is far better to use a dam.

As frequent reference will be made to the use of the Gates-Glidden drills, it may not be amiss to say a few words as to the proper way to use them. We will all agree that they are not to be used except with care, never forced, and frequently withdrawn and cleaned; the pulp canal, as well, must be cleaned, so that no debris is forced through the apex. Whether used in the straight hand piece or right angle, there should be a large, a medium, and a small drill. Which shall we use first? The advertisements usually say the smallest, following with the next in size, until you have made the canal large enough. Now, do just the reverse, and you will break fewer drills, use them more and to better advantage. Why? Because the canal or root as it leaves the pulp chamber is largest just there and will stand more reaming. There is nothing to bind the largest drill at this point; then, after the first cut, there is a sufficiency of room in which to work the next size without binding. Do not think it necssary always to drill out a canal to the apex; sometimes to merely countersink or funnel out the mouth of the canal is all that is required. If the canal needs much reaming out, find direction and length by using a very fine plain broach or explorer. The size of the crown may indicate the size and length of root, but from careful study and examination of large numbers of teeth the frequent fallacy of this is seen.

When a broach is passed through the apex of a root the sensation experienced is like a pin prick, while if it touches a portion of nerve in the canal it will be more acute. Sometimes a broach so fills the canal that it acts like a piston, and air and debris are forced through. This causes pain. A more slender broach will pass much further with no pain.

To begin with the simpler and most easily entered tooth, we will first consider the superior incisors. Usually there is a filling or cavity on either the mesial or distal side, rarely one of sufficient depth on the labial. If a filling, and a good one, leave it intact and open on the lingual surface in the fossa. If a cavity, and of such shape and size as will with slight enlargement permit the passage of broaches and other instruments directly into the canal, its orifice will be sufficient.

( *To be Continued.* )

---

Lunar Caustic with Cocain.—To make the application of silver nitrate in sensitive places less painful, simultaneous use of cocain nitrate is recommended. The hydrochloride is not suitable for this, as it precipitates silver as chloride.—*Ztsch. f. Ph.*

---

Plaster of Paris Impressions. —Let the patient thoroughly rinse out the mouth with a little milk immediately before the tray is inserted instead of using vaselin or glycerin, either of which is objectional to many patients. —*H. W. Greenfield, Ash's Quarterly.*

